# Decreased Peritoneal Ovarian Cancer Growth in Mice Lacking Expression of Lipid Phosphate Phosphohydrolase 1

**DOI:** 10.1371/journal.pone.0120071

**Published:** 2015-03-13

**Authors:** John Nakayama, Timothy A. Raines, Kevin R. Lynch, Jill K. Slack-Davis

**Affiliations:** 1 Department of Obstetrics and Gynecology, The Cancer Center, University of Virginia, Charlottesville, Virginia, United States of America; 2 Department of Microbiology, Immunology and Cancer Biology, The Cancer Center, University of Virginia, Charlottesville, Virginia, United States of America; 3 Department of Pharmacology, The Cancer Center, University of Virginia, Charlottesville, Virginia, United States of America; 4 The Cancer Center, University of Virginia, Charlottesville, Virginia, United States of America; Cincinnati Children's Hospital Medical Center, UNITED STATES

## Abstract

Lysophosphatidic acid (LPA) is a bioactive lipid that enhances ovarian cancer cell proliferation, migration and invasion *in vitro* and stimulates peritoneal metastasis *in vivo*. LPA is generated through the action of autotaxin or phospholipases, and degradation begins with lipid phosphate phosphohydrolase (LPP)-dependent removal of the phosphate. While the effects of LPA on ovarian cancer progression are clear, the effects of LPA metabolism within the tumor microenvironment on peritoneal metastasis have not been reported. We examined the contribution of lipid phosphatase activity to ovarian cancer peritoneal metastasis using mice deficient in LPP1 expression. Homozygous deletion of LPP1 (LPP1 KO) results in elevated levels and decreased turnover of LPA *in vivo*. Within 2 weeks of intraperitoneal injection of syngeneic mouse ovarian cancer cells, we observed enhanced tumor seeding in the LPP1 KO mice compared to wild type. However, tumor growth plateaued in the LPP1 KO mice by 3 weeks while tumors continued to grow in wild type mice. The decreased tumor burden was accompanied by increased apoptosis and no change in proliferation or angiogenesis. Tumor growth was restored and apoptosis reversed with exogenous administration of LPA. Together, these observations demonstrate that the elevated levels of LPA per se in LPP1 KO mice do not inhibit tumor growth. Rather, the data support the notion that either elevated LPA concentration or altered LPA metabolism affects other growth-promoting contributions of the tumor microenvironment.

## Introduction

Lysophsphatidic acid (LPA) is a bioactive lipid that regulates several cellular functions critical for tumorigenesis and metastasis including proliferation, survival, cytoskeletal reorganization, migration, invasion and cytokine production [1–5]. The importance of LPA to ovarian cancer progression was established when it was identified as a growth factor in malignant ascites [6]. LPA stimulates cellular activities via at least three (LPA1, LPA2, and LPA3) and perhaps as many as 6 (LPA4–6) G-protein coupled receptors. LPA1 is expressed on normal ovarian surface epithelium; the expression of LPA2 and LPA3 is induced in the cancer cells [2]. Upon binding its receptor, LPA stimulates ovarian cancer cell proliferation through activation of Gα12 [4]. All three receptors regulate ovarian cancer cell migration and invasion directly by activating pro-migratory Rac and Rho-dependent signaling pathways [1,7]. In addition, LPA promotes ovarian cancer growth and metastasis indirectly by stimulating the production of proteases (MMP and urokinase plasminogen activator) [8,9] and cytokines (IL-6 and IL-8), which play a role in ovarian cancer invasion and metastasis [10]. LPA binding to LPA2 or LPA3 increases production of IL-6, IL-8, and VEGF. Indeed, knockdown of LPA2 or LPA3 decreases IL-6 production, and their over-expression leads to increased serum levels of IL-6 and VEGF, increased tumor burden and shortened survival times in a mouse model of ovarian cancer peritoneal metastasis; modulation of LPA1 had no significant effect in this study [11].

LPA is produced by a variety of cells within the tumor microenvironment including platelets, mesothelial cells, adipocytes, endothelial cells and ovarian cancer cells [12,13], and in the absence of cancer, concentrations are tightly maintained below 1 μM. Levels of LPA are significantly elevated in plasma and ascites (up to 50 μM) of women with ovarian cancer [14], and increased plasma LPA has been suggested as a biomarker for ovarian cancer [15]. Members of the phospholipase A_1_ (PLA_1_) and PLA_2_ families remove a fatty acid chain from phosphatidic acid to form LPA. Autotaxin (ATX), an extracellular lysophospholipase D also generates LPA following the removal of choline from lysophosphatidylcholine. PLA_2_ and autotaxin are elevated in ovarian cancer patients [16,17], and a positive feedback loop exists between vascular endothelial growth factor (VEGF) and ATX production by ovarian cancer cells [18].

LPA catabolism is initiated by lipid phosphate phosphohydrolases (LPPs), types 1, 2 and 3, which remove the phosphate to generate monoacylglycerol (MAG). MAG is further cleaved by monoacylglycerol lipase to release the fatty acid chain from glycerol. LPP1 and LPP3 expression is reduced in human ovarian cancers relative to normal ovarian tissue [19], while forced over-expression of either LPP1 or LPP3 decreases tumorigenesis of ovarian cancer cells in mouse models presumably from decreased levels of LPA [19,20].

In addition to regulating ovarian cancer cell proliferation, survival, migration and invasion *in vitro*, the ability of LPA to promote ovarian cancer invasion and growth has been demonstrated in mouse models of peritoneal metastasis; daily injection or implantation of pumps producing high concentrations of LPA increased tumor burden in immune compromised and syngeneic mouse models [21,22]. However, the effects of LPA metabolism within the tumor microenvironment on peritoneal metastasis have not been reported. We sought to determine whether impaired LPA phosphatase (specifically LPP1) activity affected ovarian cancer peritoneal metastasis. Rather than target LPP1 activity in the tumor cells, we examined the effect of LPP1 loss in the tumor microenvironment using mice lacking LPP1 expression following the insertion of an exon-trap (LPP1 KO) [23] and syngeneic mouse ovarian cancer cells [24]. Lipid phosphatase activity in LPP1 KO mice is markedly decreased (35–95%) in multiple tissues, including those found within the peritoneal cavity. Additionally, plasma LPA is metabolized 4 times more slowly than wild type mice, and plasma concentrations of LPA are significantly elevated in the LPP1 KO mice [23]. Here, we report that peritoneal ovarian cancer growth in LPP1 KO mice was elevated as early as 2 weeks after initiation relative to wild type controls; however, while tumor burden continued to increase in wild type mice, it plateaued after 3 weeks in mice lacking LPP1 expression. The decreased tumor burden was accompanied by increased apoptosis and no change in proliferation or angiogenesis. Data from a matrigel plug angiogenesis assay did not uncover defects in LPP1 KO mice. Furthermore, exogenous administration of LPA stimulated ovarian cancer growth to an equal extent in wild type and LPP1 KO mice. Together, these observations support the notion that LPP1 KO mice either lack a critical growth-promoting factor or produce an inhibitor of ovarian cancer growth, which can be overcome by increasing LPA following exogenous administration.

## Materials and Methods

### Cell Culture and Transfection

ID8 cells were generously provided by Dr. K. Roby (University of Kansas) [24] and cultured in DMEM supplemented with 4% fetal bovine serum, 100 units/mL penicillin, 100 μg/mL streptomycin, 5 μg/mL insulin, 5 μg/mL transferrin, and 5 ng/mL sodium selenite. They were passaged twice through C57BL/6 mice to increase tumor take and decrease growth kinetics. The resultant cell line, ID8ip2, was transduced with lentivirus expressing luciferase (GeneCopoeia, Rockville, MD), and stably expressing populations (ID8ip2Luc) were obtained following puromycin (2 μg/mL) selection for 2 weeks.

### Mouse ovarian cancer peritoneal metastasis

All animal experiments were performed following approval from the Institutional Animal Care and Use Committee at the University of Virginia. Six to 8 week-old female wild type (C57BL/6) or LPP1 hypomorphic (LPP1 KO) mice were injected intraperitoneally (IP) with 10^6^ ID8ip2Luc cells in 200 μL PBS. Experiments were performed with groups of 3–5 mice per genotype and/or treatment; the total number of mice analyzed (n) is indicated in the figure legend. Mice were observed 2–3 times per week by laboratory personnel and monitored for signs of distress (i.e., changes in appearance, respiration, activity, etc.) and weighed; mice showing signs of distress or losing greater than 15% body weight were euthanized and examined for tumor. LPA (18:1, Sigma-Aldrich) was suspended in 0.5% fatty acid-free bovine serum albumin in phosphate-buffered saline (PBS) at a concentration of 100 μmol/L, and 200 μL were delivered IP once daily beginning 1 day after tumor initiation and continued for 6 weeks. Daily administration of vehicle served as negative control. Tumor burden was monitored weekly by measuring light emission following IP luciferin administration as an indication of luciferase activity using an IVIS imaging system (Molecular Imaging Core, University of Virginia). Total flux (photons/sec) was determined for the entire abdominal cavity. Upon experimental termination, mice were euthanized and tumor burden evaluated upon necropsy by counting the number of tumor nodules, and weighing the omentum (primary site of tumor implantation) and any additional tumor nodules. Formalin-fixed, paraffin-embedded tissues were sectioned and H&E stained (University of Virginia Research Histology Core) to evaluate microscopic tumor burden, the extent of peritoneal invasion, and mitotic index.

### Immunohistochemistry

One section of tumor containing omentum per mouse was subjected to immunohistochemistry for Ki67 (1:500, rabbit monoclonal, Epitomics, Cat. # 4203–1), cleaved caspase 3 (1:500, rabbit polyclonal, Cell Signaling, Cat. # 9661), CD31 (endothelial cell; 1:4, rabbit polyclonal, Abcam, Cat. # ab28365), or VCAM-1 (1:800, rabbit polyclonal, Abcam, Cat. # ab134047) (University of Virginia Biorepository Tissue Research Facility). Briefly, sections were boiled in low pH (Ki67, CD31) or high pH (cleaved caspase 3, VCAM-1) EnVision FLEX Target Retrieval Solution (Dako) and stained with the indicated concentrations of each antibody. Antigen–antibody complex was detected using Envision Dual Link (Dako) followed by incubation with 3,3’-diaminobenzidine tetrahydrochloride (DAB+) chromogen (Dako) and counterstained with hematoxylin. Ki67, and cleaved caspase 3 staining was quantified by summing the number of positively and negatively stained tumor cells in 5 high-powered (400X magnification) fields per section (one section per mouse) to calculate the percent positive cells. The extent of vessel formation was determined by counting the number of CD31 stained vessels and normalizing it to the total tumor area (outlined and calculated using ImageJ) per 200X field (5 fields per section, 1 section per mouse). The percentage of mesothelial cells expressing membranous VCAM-1 was scored positive at >50%.

### Matrigel plug angiogenesis assay

Growth factor-reduced matrigel (BD Biosciences) was mixed with ID8ip2Luc cell conditioned media (1:1) or with 1 μg/ml FGF, 20 μg/ml VEGF [25] and injected subcutaneously into 10 wild type or 8 LPP1 KO mice. Each mouse received 1 matrigel plug with conditioned media and one with FGF/VEGF. After 10 days, the mice were euthanized, and matrigel plugs were recovered, fixed in 10% formalin, embedded, sectioned, and stained for CD31 to mark vessels. The total number of CD31 positive vessels in five 200X magnification fields per plug was counted. Plugs were not recovered from all mice; the number of plugs for each genotype and angiogenic stimulus is indicated in the figure legend.

### Statistical Analysis

All data were analyzed using GraphPad Prism 6 software. Luminescence data were analyzed using 2-way analysis of variance (ANOVA) followed by Tukey’s multiple comparisons test. One-way ANOVA followed by Tukey’s multiple comparisons test was used to analyze omentum weights and tissue staining from mice treated with LPA or vehicle. The incidence of invasive tumors or VCAM-1 staining was analyzed using Fisher’s Exact test. The remaining data were analyzed with an unpaired t-test or the Wilcoxan-Mann-Whitney test depending on whether the data were parametric.

## Results

### Increased tumor seeding of the peritoneum in LPP1 KO mice

**Fig 1:**
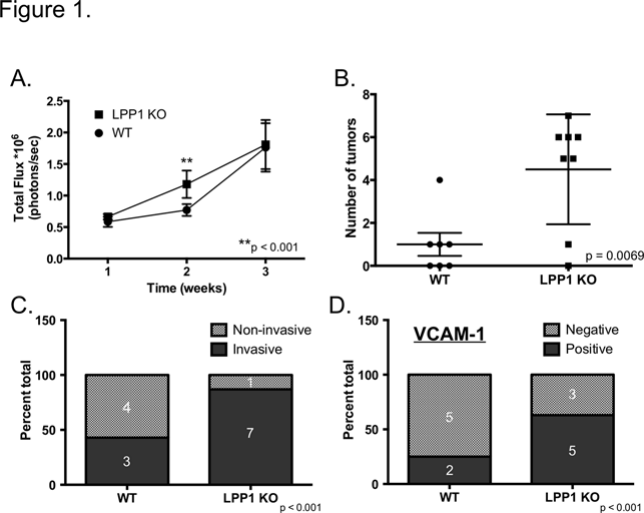
Increased peritoneal tumor seeding in LPP1 KO mice. ID8ip2Luc ovarian cancer cells were injected IP into C57BL/6 (WT) or LPP1 KO mice. **(A)** Mice (WT n = 10; LPP1 KO n = 12) were imaged weekly following tumor initiation to monitor tumor growth. Data represent mean total flux (photons/second) ± std err and were analyzed by 2-way ANOVA followed by Tukey’s multiple comparisons test. **(B)** The total number of microscopic tumor nodules was counted in an individual, randomly selected, H&E stained section of omentum per mouse obtained 2 weeks after tumor initiation (WT n = 7; LPP1 KO n = 8). Data represent mean ± std err; p = 0.0069 determined by Student’s t-test. **(C)** The percentage of wild type or LPP1 KO (from **(B)**) mice with invasive (solid bars) or non-invasive (includes no tumor; stippled bars) tumors 2 weeks after tumor initiation. Number of mice per outcome is indicated. Significance determined by Fisher’s Exact Test. **(D)** The percentage wild type or LPP1 KO (from **(B)**) mice with (positive, solid bars) or without (negative, stippled bars) mesothelial VCAM-1 staining by IHC 2 weeks after tumor initiation. Number of mice per outcome is indicated. Significance determined by Fisher’s Exact Test.

While the contribution of LPA to ovarian cancer growth and progression has been studied extensively [1,2,4,7–11], the effects of LPA metabolism on cancer progression are poorly understood. We investigated the effect of reduced LPA turnover on metastatic peritoneal ovarian cancer growth and progression using mice lacking the lipid phosphatase, LPP1 (LPP1 KO) and a syngeneic mouse ovarian cancer cell line, ID8ip2Luc. *In vivo* imaging of mice following intraperitoneal (IP) injection of ID8ip2Luc cells revealed increased luminescence in LPP1 KO mice compared to wild type within 2 weeks (Fig. 1A). Physical examination of the peritoneal cavity upon necropsy 2 weeks after tumor initiation showed no indication of tumor nodules (data not shown) and no difference in weights of the omentum (primary site of tumor metastasis) between genotypes (S1 Fig.) or from non-tumor bearing mice (data not shown). However, evaluation of H&E stained sections of the omentum revealed the presence of microscopic tumor nodules (S1 Fig.), consistent with previous observations [26]. Importantly, significantly more microscopic nodules were observed in omentums from LPP1 KO mice compared to wild type (Fig. 1B). Furthermore, LPP1 KO mice were more likely to harbor invasive lesions (Fig. 1C, S1 Fig.) that coincided with mesothelial VCAM-1 expression (Fig. 1D, S1 Fig.), which promotes tumor invasion of the mesothelium [26]. Together, these observations indicate that loss of LPP1 in the tumor microenvironment facilitates tumor seeding and mesothelial invasion of the peritoneal cavity.

### Diminished tumor growth and increased apoptosis in LPP1 KO mice

**Fig 2:**
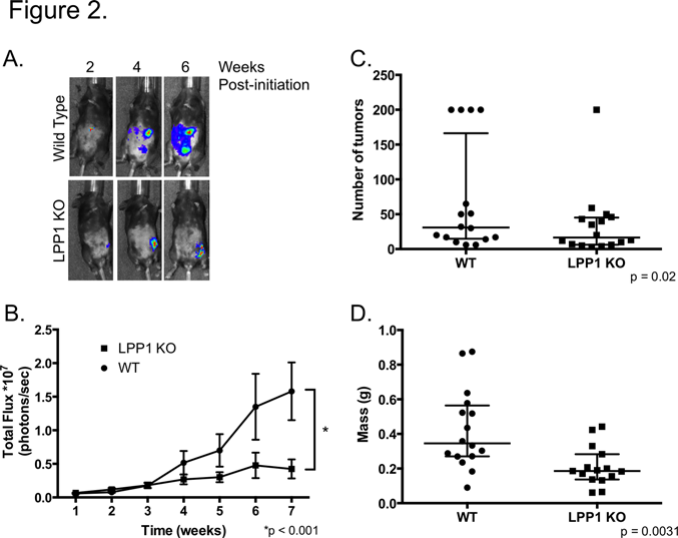
Peritoneal ovarian cancer growth is reduced in LPP1 KO mice. Following IP injection of ID8ip2Luc cells, mice were imaged weekly to monitor tumor growth kinetically. **(A)** Representative luminescence images of wild type and LPP1 KO mice with tumor taken at 2-week intervals. **(B)** Quantification of total flux (photons/second) was determined weekly for wild type (n = 10) and LPP1 KO (n = 12) mice. Data represent mean ± std err; p < 0.001 determined by 2-way ANOVA followed by Tukey’s multiple comparisons test. **(C**) Data represent the number of macroscopic tumor nodules per mouse determined 8 weeks after tumor initiation. Mice with too many nodules to count were assigned a value of 200. Data represent median with 25^th^ and 75^th^ percentiles; p = 0.02 determined using the Wilcoxan-Mann-Whitney test. **(D)** Tumor volume was determined by weighing the omentum (primary site of tumor formation) and all associated tumor nodules 8 weeks after tumor initiation; each point indicates an individual mouse. Data represent median with 25^th^ and 75^th^ percentiles; p = 0.0031 determined by Student’s t-test.

**Fig 3:**
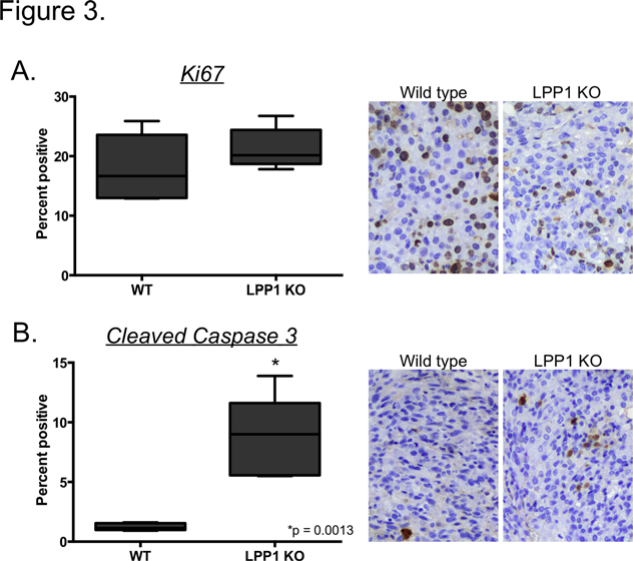
Increased apoptosis in tumors from LPP1 KO mice. Tumors collected 8 weeks after initiation were sectioned and stained for Ki67 **(A)** or cleaved caspase 3 **(B). S**taining was quantified by counting the number of positive and negative cells from 5 high-powered fields (400x magnification) of tumor per mouse, 5 mice per group. The data are presented as percent positive. Box and Whiskers plots show minimum, 25^th^ percentile, median, 75^th^ percentile and maximum values. *p < 0.001, Student’s t-test.

While the microenvironment of LPP1 KO mice favors tumor implantation in the peritoneal cavity, wild type mice showed equivalent tumor burden indicated by luminesence within 3 weeks of initiation (Fig. 1A). More strikingly, robust tumor growth ensued in wild type mice as shown by increased luminescence over time while tumors plateaued in the LPP1 KO mice at 3 weeks (Fig. 2A and 2B). Additionally, wild type mice had increased numbers of macroscopic tumor nodules studding the peritoneal cavity and increased tumor volume determined by weighing the omentum and associated tumors (Fig. 2C and 2D). To determine whether the lack of tumor growth in the LPP1 KO mice was due to less proliferation or increased apoptosis, tumors were stained for the proliferative marker Ki67 or cleaved caspase 3, as a marker of apoptosis. Tumors derived from wild type and LPP1 KO mice had equivalent levels of proliferation (Fig. 3A). However, tumors from LPP1 KO mice had 3 times more cells staining positively for cleaved caspase 3 compared to wild type (Fig. 3B) indicating a significant increase in apoptosis. Therefore, while loss of LPP1 in the tumor microenvironment facilitates tumor seeding, it is insufficient to sustain tumor growth over time due at least in part to an increase in apoptosis.

### Angiogenesis is not altered in LPP1 KO mice

**Fig 4:**
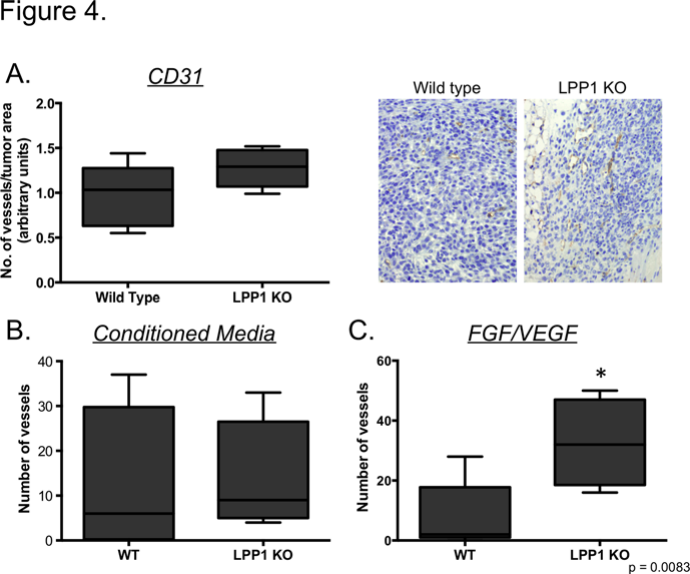
Angiogenesis is not defective in LPP1 KO mice. **(A)** Tumors from mice 8 weeks after initiation were sectioned and stained for CD31, and the total number of CD31 positive vessels per tumor area from 5 high powered fields (hpf; 200X magnification) per mouse for wild type (WT, n = 5) and LPP1 KO (n = 5) mice is plotted. The total number of CD31 positive vessels observed in 5 hpf of matrigel plugs containing conditioned media from ID8ip2Luc cells **(B)** or FGF/VEGF **(C)** is plotted (see Materials and Methods). Data are presented with Box and Whiskers plots as described for Fig. 3. *p = 0.0083, Student’s t-test.

Increased apoptosis in LPP1 KO mice could occur as a result of a lack of nutrients supplied to the tumor due to defective angiogenesis, the absence of a pro-survival factor or the presence of an inducer of cell death. Indeed, mice lacking LPP3 expression are embryonic lethal due to a defect in angiogenesis [27]. To determine whether angiogenesis was defective in LPP1 KO mice, tumors were stained for the endothelial marker CD31. As shown in Fig. 4A, tumors from wild type and LPP1 KO mice had similar levels of CD31 staining. To more objectively evaluate whether there was an intrinsic alteration in angiogenesis, we performed a matrigel plug assay. Briefly, matrigel embedded with tumor cell conditioned media or FGF and VEGF was implanted into the flanks of wild type or LPP1 KO mice to stimulate angiogenesis (S2 Fig.). Tumor cell conditioned media stimulated an equivalent number of vessels in wild type and LPP1 KO mice (Fig. 3B). FGF/VEGF stimulated vessel formation to a greater extent in LPP1 KO mice compared to wild type (Fig. 3C). Together, these observations indicate that the increased apoptosis and accompanying decrease in tumor burden in the LPP1 KO mice was not due to a defect in angiogenesis.

### Exogenous growth factor administration overcomes tumor suppression in LPP1 KO mice

**Fig 5:**
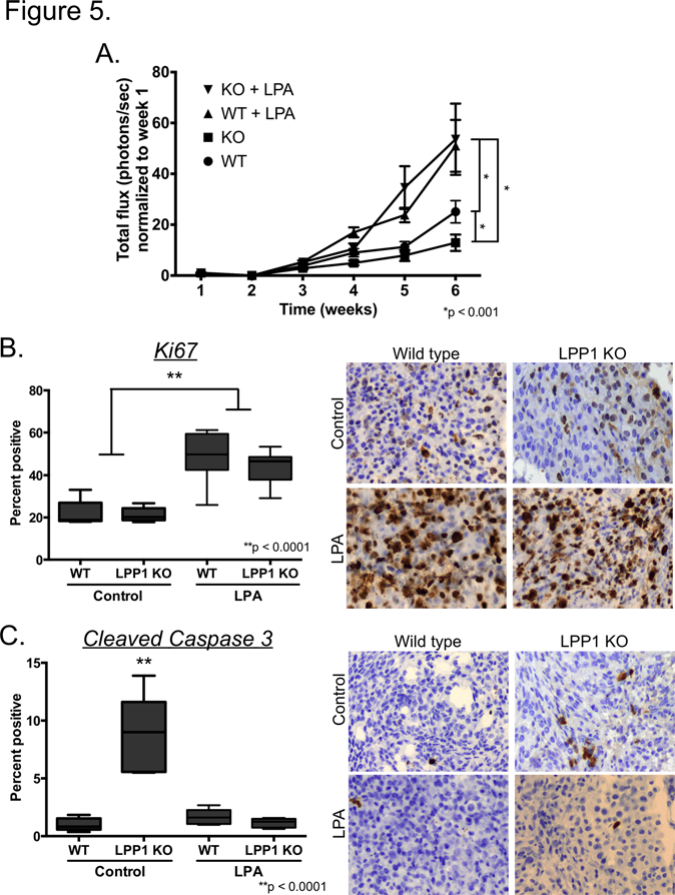
Daily injection of LPA rescues tumor suppression and stimulates ovarian cancer growth. ID8ip2Luc ovarian cancer cells were injected IP into wild type (WT, n = 19) or LPP1 KO (n = 20) mice. Mice received daily injections of LPA (n = 6 WT, n = 7 LPP1 KO) or vehicle (n = 13 for both genotypes) beginning the day after tumor injection. **(A)** Mice were imaged weekly following tumor initiation to monitor tumor growth. Data represent mean total flux (photons/second) ± std err; *p < 0.001, 2-way ANOVA followed by Tukey’s multiple comparisons test. Tumors were collected 6 weeks after initiation, sectioned and stained for Ki67 **(B)** or cleaved caspase 3 **(C).** Quantification of Ki67 and cleaved caspase 3 was achieved by determining the percentage of positively stained cells in 5 high-powered fields (400x magnification) per mouse, 5 mice per group. Data are presented with Box and Whiskers plots as described for Fig. 3. **p < 0.0001, 2-way ANOVA followed by Tukey’s multiple comparisons test.

Reduced tumor growth in the absence of defects in angiogenesis is indicative of the lack of a growth-promoting environment in the LPP1 KO mice. Such an environment could involve a multitude of components including the lack of a growth-promoting (pro-survival) factor, the presence of an inhibitor of growth (inducer of cell death), the inability to recruit auxiliary cells necessary to promote growth, inappropriate cell-cell interactions within the tumor or any combination of these or additional effects. We hypothesized that exogenous administration of high concentrations of a growth factor would override the negative consequences of the growth inhibitory environment. LPA is a well-established stimulator of ovarian cancer cell growth [2,4]. Daily administration of LPA has been reported to stimulate growth of human ovarian cancer cells in immune compromised mice [21] and tumors in the ID8 mouse ovarian cancer model [22]. Therefore, we tested the ability of LPA to stimulate ID8ip2Luc tumor growth in LPP1 KO compared to wild type mice. Daily administration of LPA significantly increased tumor growth in wild type mice starting 4 weeks after initiation and culminated in 3 times more luminescence compared to wild type mice without exogenous LPA (Fig. 5A), consistent with previous observations [22]. LPA also stimulated tumor growth in LPP1 KO mice to the same extent observed in wild type mice treated with LPA (Fig. 5A). In both cases, LPA increased tumor cell proliferation (Fig. 5B). Strikingly, LPA decreased the number of cells that stained positive for cleaved caspase 3 to the level found in tumors from wild type mice with or without LPA (Fig. 5C). The data indicate that delivery of increased concentrations of LPA reverses the negative growth environment found in LPP1 KO mice.

## Discussion

LPA is an important regulator of ovarian cancer growth and metastasis. In addition to the increased levels found in ascites, which is accompanied by increased ATX activity, the tumors themselves have de novo expression of LPA receptors (LPA2 and LPA3) [2] and decreased expression of LPP1 and LPP3 [19,20]. Here, we examined the contribution of LPA metabolism in non-cancer cells within the tumor microenvironment on the establishment and growth of ovarian cancer within the peritoneal cavity. Loss of LPP1 expression within the tumor microenvironment led to increased tumor seeding following IP injection of ovarian cancer cells; however, subsequent growth was hampered, which was accompanied by increased apoptosis and no defects in tumor cell proliferation, angiogenesis or macrophage and lymphocyte recruitment (data not shown). These observations indicate that loss of LPP1 creates an environment insufficient to support tumor growth perhaps due to the lack of pro-survival factors or the presence of growth inhibitors.

LPP1 KO mice are characterized as having increased plasma LPA levels and reduced LPA turnover [23]. LPA stimulates many aspects of ovarian cancer cell biology including proliferation, survival, migration and invasion [2,4]. Indeed, we observed an increase in mesothelial invasion and tumor seeding early after tumor initiation in LPP1 KO mice. LPA has been reported to stimulate mesothelial invasion in cell culture models [13] and *in vivo* [22], although a mechanism has not been defined. Previously, we demonstrated a role for VCAM-1 expressed on the mesothelium in the regulation of ovarian cancer invasion in vitro and in vivo [26]. Interestingly, LPP1 KO mice had a higher incidence of mesothelial VCAM-1 expression offering the possibility that LPA might regulate VCAM-1 expression to promote mesothelial invasion. Additional studies are necessary to determine whether this is the case.

While LPP1 KO mice have increased tumor seeding in the presence of elevated levels and reduced turnover of LPA, subsequent tumor growth was stunted and accompanied by increased apoptosis, a result that contradicts well-established pro-growth, pro-survival effects of LPA. The mechanisms by which loss of LPP1 in the tumor microenvironment halts tumor growth are unclear. One possibility is that elevated LPA as a result of LPP1 deficiency affects other cellular components of the tumor microenvironment to negatively impact tumor growth. The tumor microenvironment contains a complex mixture of cytokines, growth factors and multiple cell types, including endothelial and immune cells that function together to promote tumor growth [28]. While the increased apoptosis in tumors from LPP1 KO mice could be due to the lack of nutrient supply to the tumor, we observed no defect in angiogenesis. LPA has been reported to stimulate migration and survival of macrophages [29–31], facilitate transmigration of lymphocytes [29,32] and inhibit the cytotoxic activity of T cells and NK cells [33,34], all of which would be expected to promote tumor growth. We found no difference in the numbers of macrophages or lymphocytes (B, T and NK) in tumors from wild type and LPP1 KO mice (data not shown). However, it is possible that tumors from LPP1 KO mice have different subsets of immune cells (i.e., the lack of pro-tumor or overrepresentation of anti-tumor) and/or altered regulation of their activity.

The effects of loss of LPP1 on tumor growth could be due to alterations in the metabolism of other phosphoglycerides. In addition to LPA, LPP1 acts on other phosphoglycerides including phosphatidic acid and sphingosine 1-phosphate (S1P) [35], which at high concentrations induces ovarian cancer cell death [36]. However, we observed no differences in S1P plasma concentration between wild type and LPP1 KO mice (data not shown). The concentration of other phosphoglycerides has not been measured. It is important to consider that the turnover of LPA and/or other phosphoglycerides might be equally important as their absolute concentration.

Together, the data presented here indicate that the loss of LPP1 creates an environment insufficient to support tumor growth. Exogenous supply of higher concentrations of LPA was able to overcome the inhibitory environment to stimulate tumor growth and diminish apoptosis demonstrating that LPA stimulates tumor cell growth regardless of any potential negative effects created by the loss of LPP1 expression. Further investigation of the mechanisms (including potential cellular targets) responsible for reduced tumor growth in the absence of LPP1 would provide additional important information regarding the effects of phosphoglycerides and their metabolism on the tumor microenvironment as well as components within the microenvironment that might be important in promoting ovarian cancer progression.

## Supporting Information

S1 Fig Characterization of tumors and VCAM-1 expression in LPP1 KO and wild type mice 2 weeks after tumor initiation. **(A)** Omentums were removed and weighed 2 weeks after tumor initiation from wild type and LPP1 KO mice. Each point indicates a single individual, and the data represent the mean ± std err. **(B)** H&E stained sections of omentum were evaluated for microscopic tumors and the extent of invasion. Arrows indicate the mesothelium; arrowheads indicate tumor. Non-invasive tumors were characterized as having a smooth interface between the tumor and underlying tissue. Invasive tumors were scored as those spidering through and taking over the underlying tissue, in this case, omental fat. **(C)** Omentums from wild type (top panel) and LPP1 KO (bottom panel) mice were obtained 2 weeks after tumor initiation and stained for VCAM-1 expression using IHC (see Materials and Methods). Representative images depict the mesothelium (arrows) and positive VCAM-1 staining (arrowheads) in the LPP1 KO mice with the lack of VCAM-1 reactivity on the mesothelium of wild type mice.
(TIF)

S2 Fig Representative images of the matrigel plug assay. Representative images of CD31 positive vessels (indicated by arrows) in matrigel plugs containing conditioned media from ID8ip2Luc cells **(A)** or FGF/VEGF **(B)** are shown.
(TIF)
