# Supplementary figures and images for: Decreased Peritoneal Ovarian Cancer Growth in Mice Lacking Expression of Lipid Phosphate Phosphohydrolase 1
Source: PLoS One. 2015 Mar 13;10(3):e0120071. doi: 10.1371/journal.pone.0120071 (PMC4359083; doi:10.1371/journal.pone.0120071)

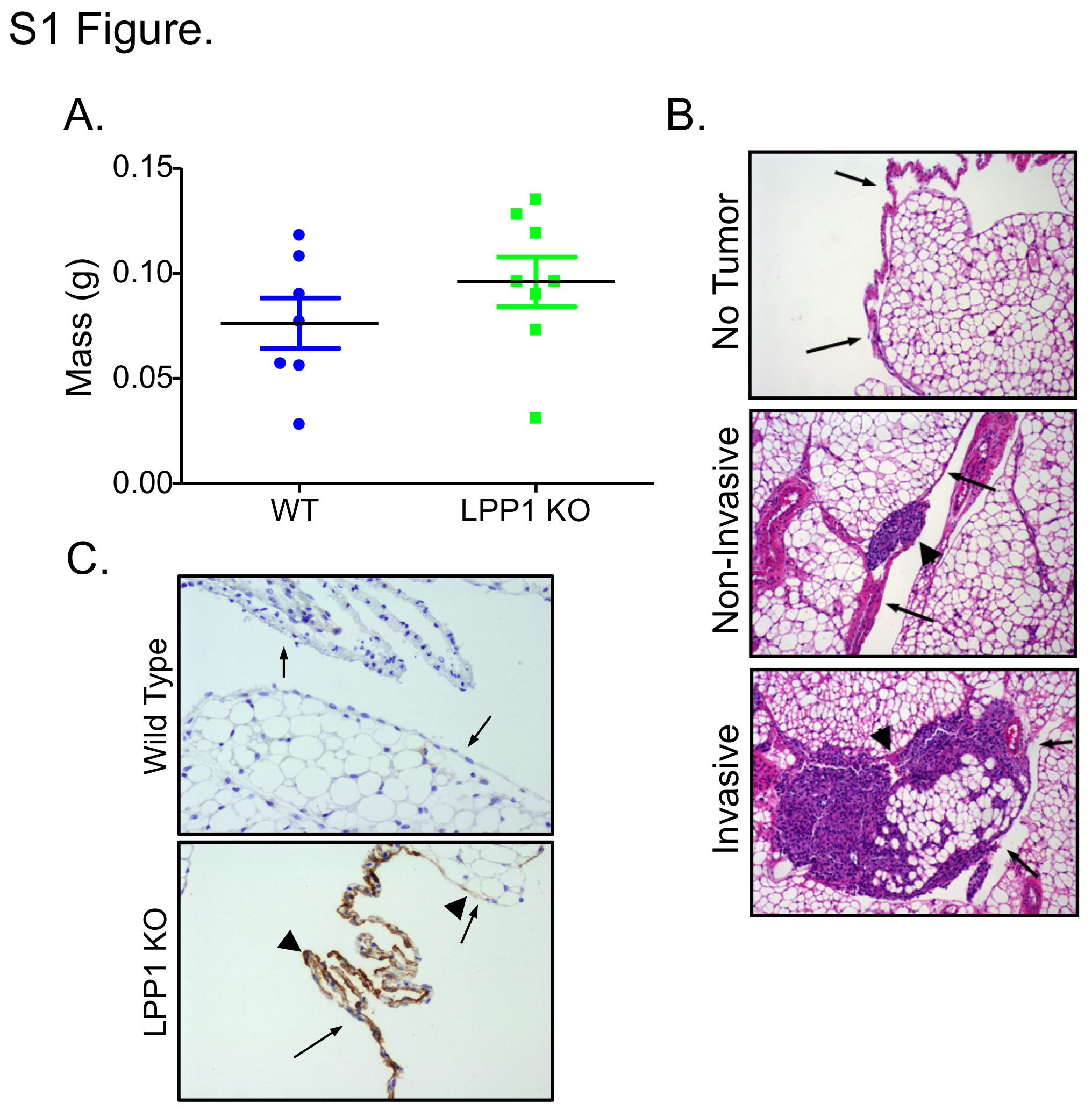

Supplement: S1 Fig — (A) Omentums were removed and weighed 2 weeks after tumor initiation from wild type and LPP1 KO mice. Each point indicates a single individual, and the data represent the mean ± std err. (B) H&E stained sections of omentum were evaluated for microscopic tumors and the extent of invasion. Arrows indicate the mesothelium; arrowheads indicate tumor. Non-invasive tumors were characterized as having a smooth interface between the tumor and underlying tissue. Invasive tumors were scored as those spidering through and taking over the underlying tissue, in this case, omental fat. (C) Omentums from wild type (top panel) and LPP1 KO (bottom panel) mice were obtained 2 weeks after tumor initiation and stained for VCAM-1 expression using IHC (see Materials and Methods). Representative images depict the mesothelium (arrows) and positive VCAM-1 staining (arrowheads) in the LPP1 KO mice with the lack of VCAM-1 reactivity on the mesothelium of wild type mice. (TIF) [file pone.0120071.s001.tif]

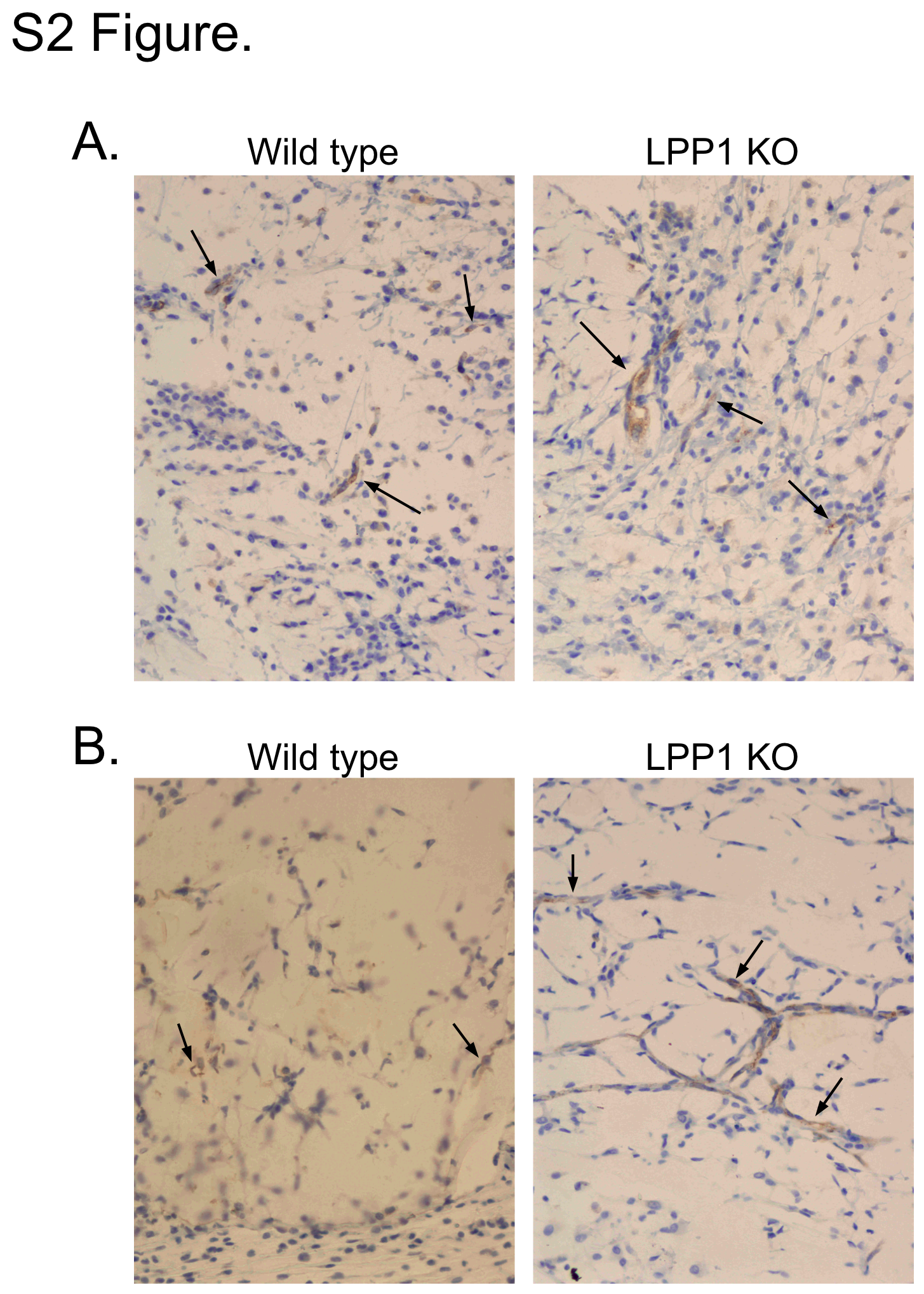

Supplement: S2 Fig — Representative images of CD31 positive vessels (indicated by arrows) in matrigel plugs containing conditioned media from ID8ip2Luc cells (A) or FGF/VEGF (B) are shown. (TIF) [file pone.0120071.s002.tif]
